# A Japanese family with dystonia due to a pathogenic variant in *SGCE*

**DOI:** 10.1038/s41439-022-00207-8

**Published:** 2022-08-22

**Authors:** Takuya Morikawa, Shiroh Miura, Luoming Fan, Emina Watanabe, Ryuta Fujioka, Hiromichi Motooka, Shingo Yasumoto, Yusuke Uchiyama, Hiroki Shibata

**Affiliations:** 1 Division of Genomics, Medical Institute of Bioregulation, Kyushu University, 3-1-1, Maidashi, Higashi-ku, Fukuoka, Japan; 2 Department of Neurology and Geriatric Medicine, Ehime University Graduate School of Medicine, 454, Shitsukawa, Toon, Ehime Japan; 3 Department of Food and Nutrition, Beppu University Jounior College, 82, Kitaishigaki, Beppu, Oita, Japan; 4 Department of Neuropsychiatry, Kurume University School of Medicine, 67, Asahimachi, Kurume, Japan; 5 Department of Radiology, Kurume University School of Medicine 67, Asahimachi, Kurume, Japan

**Keywords:** Medical genomics, Neuroscience

## Abstract

Dystonia (DYT) is a heterogeneous neurological disorder, and there are many types of DYT depending on the responsible genes. DYT11 is an autosomal dominant DYT caused by functional variants in the *SGCE* gene. We examined a Japanese patient with myoclonic dystonia. By using exome analysis, we identified a rare variant in the *SGCE* gene, NM_003919.3: c.304C > T [Arg102*], in this patient. Therefore, this patient has been molecularly diagnosed with DYT11. By Sanger sequencing, we confirmed that this variant was paternally inherited in this patient. By allele-specific PCR, we confirmed that the maternally inherited normal allele of *SGCE* was silenced, and only the paternally inherited variant allele was expressed in this patient. Despite the pathogenicity, identical variants have been recurrently reported in eight independent families from different ethnicities, suggesting recurrent mutations at this mutational hotspot in *SGCE*.

Dystonia is a neurological disorder characterized by abnormal involuntary movements or postures owing to sustained or intermittent muscle contractions^1^. Monogenic forms of dystonia/dyskinesias (DYTs) are classified from DYT1 to DYT25, and 17 genes are known to be responsible for DYT^2^. In addition to these 17 genes, 9 new genes have been suggested to be responsible for DYT^3^. *SGCE* is the gene responsible for DYT11^1^.

A 19-year-old female, without a family history of involuntary movements, noticed occasional jerking in her right hand when writing or using chopsticks at the age of eight. She often had difficulty holding things in her right hand. Her symptoms were aggravated by psychological stress. Her symptoms gradually worsened and spread to her left hand. She had no other significant medical history. Neurological examination revealed no abnormalities except for action myoclonus in her hands with right dominancy and occasionally dystonic posture in her right foot during walking. Her action myoclonus was inhibited by dorsiflexion of the wrist joint. No abnormalities were observed in brain magnetic resonance imaging or electroencephalography. Her symptoms improved dramatically with clonazepam (1.5 mg/day). Her parents showed no neurological abnormalities. The patient was diagnosed with myoclonus-dystonia syndrome.

We performed exome sequencing for the patient who was diagnosed with DYT (Fig. 1A). We identified a total of 130,940 variants in the patient (II-1). Since she was diagnosed with myoclonus-dystonia syndrome^2,3^, we selected 80 variants located in 26 genes known to be associated with DYT (Table S1). Out of the 80 variants, we identified only two functional variants with a MAF (minor allele frequency) <0.02. One was a heterozygous variant in *PKRA* that is known to be associated with the autosomal recessive form of DYT, so we excluded this variant. Then, we retained only one variant, NM_003919.3:c.304C > T [Arg102*], in the epsilon sarcoglycan gene *SGCE*.

**Fig. 1:**
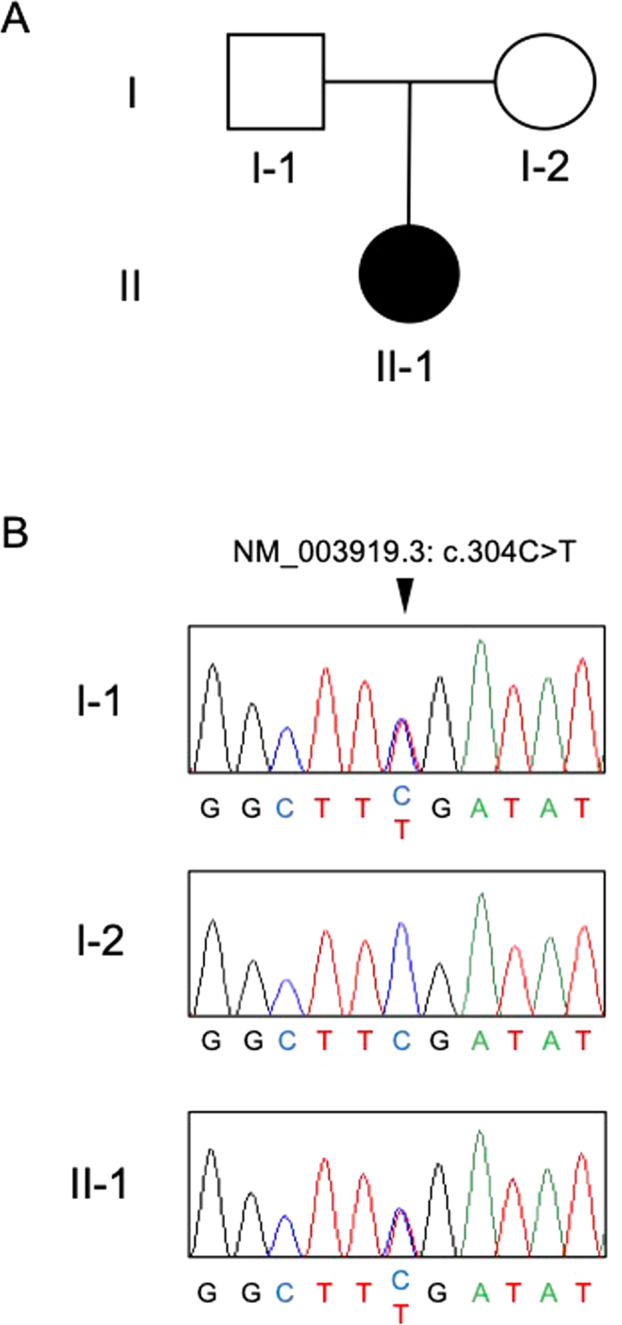
Pedigree diagram and genotyping of the *SGCE* variant. **A** Pedigree of the tested family. Squares: males, circles: females, solid symbols: affected individuals, open symbols: unaffected individuals. **B** Electropherogram of the region of the NM_003919.3: c.304C > T variant in all family members. The location of the variant is indicated by a triangle.

We validated the variant by Sanger sequencing for all family members (forward primer, 5’-ACTACCAAAGCAACATGTGTGA-3’ and reverse primer 5’-GCTTCCCCACATTTTCAGCT-3’). This variant was also found in the unaffected father (I-1) of the patient as well as the patient, indicating the paternal inheritance of this variant for this patient (Fig. 1B). *SGCE* is known to be an imprinted gene and is expressed only from paternal alleles by silencing maternal alleles^4,5^. Since the father (I-1) carrying the variant does not show DYT symptoms, it is highly likely that the variant was maternally inherited from the patient’s grandmother and was silenced in I-1 by genomic imprinting. To confirm genetic silencing, we performed allele-specific real-time qPCR using total RNA extracted from peripheral blood to determine which allele was expressed (Fig. 2A and Supplementary Table S2). Allele-specific PCR revealed that the *SGCE* mRNA of the patient (II-1) was only transcribed from the variant allele. The amount of *SGCE* mRNA in the patient (II-1) was lower than that in the mother (I-2) (Fig. 2A). This is likely due to nonsense-mediated decay degrading abnormal mRNAs. Allele-specific qPCR also revealed that the *SGCE* mRNA in the patient (II-1) was transcribed almost exclusively from the variant allele (Fig. 2B). However, we could not obtain statistical significance due to the very low expression of *SGCE* in the peripheral blood. Unfortunately, we could not include the father (I-1) in the analyses due to the unavailability of his total RNA.

**Fig. 2:**
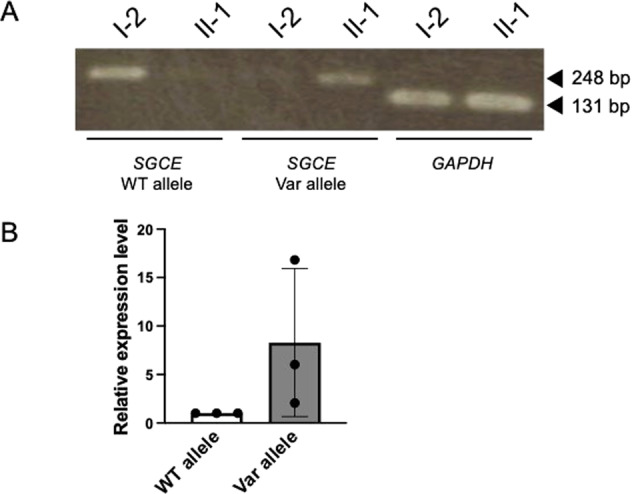
Allele-specific real-time qPCR of *SGCE*. **A** PCR products amplified with allele-specific primers were visualized by agarose gel electrophoresis. PCR products were electrophoresed on a 1.2% agarose gel at 100 V for 20 min. **B** Expression levels of the two alleles of *SGCE* in the patient (II-1) were individually examined by qPCR. qPCR using allele-specific primers was performed using Applied Biosystems 7500. The experiment was performed with three technical replications. The expression levels of the wild-type allele and variant allele are shown in open and filled columns, respectively. Values are presented as the mean ± SD. In all experiments, the expression levels from the variant alleles is shown relative to the expression level from the wild-type allele. Most of the *SGCE* mRNA of the patient expressed the variant alleles. WT wild type, Var variant.

This variant has already been identified as the variant responsible for DYT in eight independent families worldwide^6–9^. This suggests that the identical variants have recurrently arisen at the mutational hotspot in the *SGCE* gene, despite the high CADD score (CADD = 41). According to the ACMG/AMP/CAP guidelines, the variant meets the criteria of PVS1, PS1, PM1, PM2, PP1, PP3, PP4, and PP5^10^. Collectively, we concluded that the *SGCE* variant is responsible for DYT in the current patient, and she has been molecularly diagnosed with DYT11. We also directly confirmed that the genomic imprinting of *SGCE* affects the pathogenesis of DYT11. Specifically, the maternally inherited normal allele of *SGCE* was silenced, and only the paternally inherited variant allele was expressed in this patient.

## HGV database

The relevant data from this Data Report are hosted at the Human Genome Variation Database at 10.6084/m9.figshare.hgv.3213.

## Supplementary information

Supplementary Table S1

Supplementary Table S2
